# Ethical leadership and responsible initiative among Chinese university middle-level cadres: the roles of organizational fairness and identification

**DOI:** 10.3389/fpsyg.2026.2000079

**Published:** 2026-10-22

**Authors:** Lei Chen

**Affiliations:** San Niu College, Shanxi Technology and Business University, Taiyuan, Shanxi, China

**Keywords:** ethical leadership, higher education, middle-level cadres, organizational fairness, organizational identification, responsible initiative

## Abstract

Responsible initiative, the self-started and change-oriented behavior through which staff identify problems and push improvements forward, keeps universities functioning, yet the leadership conditions associated with it among middle-level cadres remain understudied. Drawing on social exchange theory and social identity theory, this cross-sectional survey proposed that ethical leadership relates to responsible initiative both directly and through two linked mechanisms: organizational fairness and organizational identification. A total of 476 middle-level cadres (64.3% male; mean age 42.5 years, SD = 6.6) from 11 universities and colleges in Shanxi Province, China, completed measures of ethical leadership (10-item Ethical Leadership Scale), organizational fairness (12 items adapted from Colquitt), organizational identification (six-item scale of Mael and Ashforth), and responsible initiative (eight items adapted from personal initiative and taking-charge instruments). Serial mediation analysis (PROCESS model 6, 5,000 bias-corrected bootstrap resamples) showed that ethical leadership was positively associated with responsible initiative (total effect = 0.389, *p* < 0.001). The indirect effect through organizational fairness [0.107, 95% CI (0.059, 0.162)], the indirect effect through organizational identification [0.086, 95% CI (0.053, 0.127)], and the serial indirect effect through fairness followed by identification [0.057, 95% CI (0.037, 0.086)] were all significant, jointly accounting for 64.2% of the total effect. These findings indicate that fair treatment and identification with the institution are parallel and sequential routes that statistically account for the association between ethical leadership and cadre initiative, and they are consistent with the value of ethics-focused leadership development and transparent appraisal procedures for strengthening responsible initiative in higher education administration. The parallel and sequential operation of exchange-based and identity-based mechanisms extends social exchange and social identity accounts of ethical leadership to a public-sector cadre population.

## 1 Introduction

Middle-level cadres occupy the connective tissue of university administration. They translate institutional strategy into school- and department-level routines, absorb demands from above while representing staff interests below, and are repeatedly identified as decisive for whether reform agendas actually reach daily operations (Wooldridge et al., 2008; Bryman, 2007). What senior leaders most need from this stratum is not passive compliance but responsible initiative: self-started, change-oriented behavior in which a cadre notices an organizational problem, works out an improvement, and persists with it despite uncertainty (Frese and Fay, 2001; Morrison and Phelps, 1999). Within the broader family of proactive behaviors, responsible initiative sits close to taking charge and personal initiative, sharing their self-starting and persistent character while remaining an extra-role, organization-directed act (Parker and Collins, 2010; Parker et al., 2010). In Chinese administrative discourse, this profile corresponds to dan dang zuo wei, taking responsibility and delivering on it, an expectation attached to cadre roles at every level. In Chinese universities, where accountability systems have tightened and cadres are evaluated on both performance and conduct, understanding the leadership conditions associated with responsible initiative has direct administrative value.

Ethical leadership, defined as the demonstration of normatively appropriate conduct through personal actions and interpersonal relationships, and the active promotion of such conduct to followers through two-way communication, reinforcement, and decision-making (Brown et al., 2005), has become one of the most intensively studied positive leadership constructs. Qualitative work traced how executives earn reputations as ethical or unethical leaders through visible choices and everyday messaging (Treviño et al., 2003), and successive reviews have consolidated the construct's nomological network (Brown and Treviño, 2006). Meta-analytic evidence links ethical leadership to follower trust, voice, organizational citizenship, and wellbeing (Ng and Feldman, 2015; Bedi et al., 2016). Field studies show that ethically led employees speak up more readily (Walumbwa and Schaubroeck, 2009), report stronger ethical climates and affective commitment (Demirtas and Akdogan, 2015), behave more prosocially (Neubert et al., 2009), and even enjoy more optimistic work teams (De Hoogh and Den Hartog, 2008). Evidence from a medical university context similarly indicates that followers' conceptions of effective leadership encompass ethical attributes such as honesty and justice, alongside respectful interpersonal behavior and context-sensitive managerial competencies (Amini et al., 2025). Recent contributions in this research topic extend the outcome set to sustainable employee wellbeing through emotional and moral resources (Chen et al., 2026) and to ethical climate and moral efficacy under team ethical leadership (Zhang et al., 2026).

Far less is known about the mechanisms that carry ethical leadership to proactive outcomes such as responsible initiative. Social exchange theory offers one account. Followers who receive considerate, fair, and principled treatment from a leader perceive an obligation to reciprocate beyond their job descriptions, and generalized exchange obligations of this kind are the classical engine of extra-role behavior (Blau, 1964; Cropanzano and Mitchell, 2005). The resource followers most visibly receive from an ethical leader is fair treatment: ethical leaders allocate outcomes equitably, apply transparent procedures, and treat people with dignity, which is precisely the content of distributive, procedural, and interactional justice (Colquitt, 2001). Justice perceptions in turn predict commitment, citizenship, and performance meta-analytically (Colquitt et al., 2001; Cohen-Charash and Spector, 2001). Fair treatment should therefore function as a first mediating route from ethical leadership to responsible initiative: cadres who experience their institution as fair acquire a reciprocity-based motive to invest discretionary effort in organizational improvement.

Social identity theory offers a second, complementary account. People derive part of their self-concept from group membership (Tajfel and Turner, 1979), and organizations are among the most consequential groups in working adults' lives (Ashforth and Mael, 1989). Organizational identification, the perceived oneness with and belonging to an organization, predicts job involvement, motivation, and supportive behavior (Mael and Ashforth, 1992; Riketta, 2005; Van Knippenberg and Van Schie, 2000). Because fair and respectful treatment signals that the institution values its members, justice judgments feed directly into identification and, through it, into cooperative engagement, as formalized in the group engagement model (Tyler and Blader, 2003). Ethical leaders, who model integrity and grant followers dignity, should strengthen identification both directly, by making membership pride-worthy, and indirectly, by making the institution feel fair. Identification should then carry this forward into responsible initiative, because cadres who see the institution's fate as their own treat its problems as their personal business.

These mechanisms have rarely been examined among university middle-level cadres in China, even though the population is distinctive. Cadre appointment and appraisal in Chinese higher education are centrally administered, promotion is competitive, and organizational citizenship carries particular weight in collectivist settings (Farh et al., 2004). Recent work in this research topic has examined university counselors' emotional exhaustion under normative commitment pressure (Cheng et al., 2026), but the middle-level administrative stratum, and its proactive conduct in particular, remains largely unmeasured. Whether ethical leadership operates on cadre initiative through fairness, through identification, or through both in sequence is an open question with theory-relevant implications for how leadership influence is transmitted in hierarchical public institutions.

Bringing the two theories together also addresses a tension that each leaves open on its own. Social exchange theory explains why fair and considerate treatment is reciprocated with discretionary effort, but it is silent on why such treatment should also bind employees to the organization itself; social identity theory explains the binding, but not the resource that sets it in motion. A sequential pathway closes this gap by specifying a conversion from exchange to identity: fairness is the evidence on which exchange obligations rest, and identification is the bond that carries those obligations into self-invested behavior. Two ordinary parallel mediators cannot detect this conversion, because a parallel specification treats the two routes as independent and leaves their ordering untested. We therefore advance, as the theoretical proposition to be tested, that the exchange-based and identity-based mechanisms linking ethical leadership to responsible initiative are not merely additive but sequentially linked, such that the fairness-based exchange route also operates through the identity route.

This study tested a serial mediation model in a sample of Chinese university middle-level cadres. We hypothesized that ethical leadership would be positively associated with responsible initiative (H1); that organizational fairness would mediate this association (H2); that organizational identification would mediate this association (H3); and that organizational fairness and identification would operate as sequential mediators along the hypothesized pathway in which ethical leadership is associated with perceived fairness, perceived fairness is associated with identification, and identification is associated with responsible initiative (H4).

## 2 Materials and methods

### 2.1 Participants

An *a priori* sensitivity check indicated that a sample of 400 provides power above 0.90 to detect standardized coefficients of 0.16 or larger in regression models with three predictors (α = 0.05, two-tailed; Cohen, 1988); recruitment exceeded this threshold. Between October 2024 and January 2025, questionnaires were distributed to and returned by 520 middle-level cadres at 11 universities and colleges in Shanxi Province, China; after the quality screening described in the Procedure section, 476 cases remained valid (91.5% of the returned questionnaires). The 11 institutions were approached through administrative channels and chosen to cover the three main higher-education sectors in the province, namely public universities, private institutions, and vocational colleges, which differ in size, ranking, and administrative structure; within each institution, participants were recruited through convenience sampling rather than random selection. Respondents ranged in age from 29 to 56 years (*M* = 42.5, SD = 6.6); 64.3% were male. Average tenure in administrative positions was 11.8 years (SD = 5.2). 28.6% held division-level posts and the remainder held section-level posts; 52.9% worked in public universities, 26.5% in private institutions, and 20.6% in vocational colleges. All participants provided written informed consent. The study protocol was approved by the Ethics Committee of Shanxi Technology and Business University and conducted in accordance with the Declaration of Helsinki.

### 2.2 Measures

Ethical leadership was measured with the 10-item Ethical Leadership Scale (Brown et al., 2005); respondents rated their direct superior on items such as “listens to what team members have to say” and “discusses business ethics or values with members.” Organizational fairness was measured with 12 items adapted from the organizational justice measure of Colquitt (2001), sampling four distributive items (outcomes reflecting effort and responsibility), four procedural items (consistency, bias suppression, accuracy, appeal), and four interactional items (treatment with dignity and adequate explanation); respondents described the outcomes, procedures, and interpersonal treatment they had experienced in their own unit during the current academic year. Sample items include “rewards reflect the effort I have invested” and “procedures are applied consistently across people.” Organizational identification was measured with the six-item scale of Mael and Ashforth (1992), including “when someone criticizes this institution, it feels like a personal insult.” Responsible initiative was measured with eight items adapted from personal initiative and taking-charge instruments (Frese and Fay, 2001; Morrison and Phelps, 1999) to the cadre setting; the items were reworded to capture the organization-directed, responsibility-taking content of dan dang zuo wei rather than change-oriented effort in general, so that the scale operationalizes the boundary drawn in the Introduction between responsible initiative and its parent constructs. Sample items are “when I notice a workable improvement, I actively pursue it even if it is not part of my formal duties” and “I take responsibility for turning problems in my unit into solutions.” All English items were translated into Chinese and back-translated for equivalence (Brislin, 1970); discrepancies were resolved by discussion among bilingual researchers. All items used a five-point scale from 1 (strongly disagree) to 5 (strongly agree), and construct scores were computed as item means. Cronbach's α values were 0.93 for ethical leadership, 0.90 for organizational fairness, 0.87 for organizational identification, and 0.89 for responsible initiative in the present sample.

### 2.3 Procedure

Questionnaires were administered on paper at administrative meetings and through a secured online platform; both channels presented the scales in a fixed order beginning with demographics. Completion took roughly 12–15 min. Participation was voluntary and anonymous, no incentive was tied to completion, and respondents were free to withdraw without consequence. All 520 returned questionnaires were screened for missing data, straight-line answering, and completion time; 44 questionnaires were excluded at this stage, leaving the 476 valid cases analyzed below. Participation was anonymous and no institution identifier was recorded; respondents are therefore treated as independent observations, potential clustering within institutions is addressed through a sensitivity analysis with institution-type indicators (reported in Results), and the remaining limitation is acknowledged in the Discussion. The study was advertised as a survey of administrative work experiences, with no reference to leadership evaluations, so as not to prime socially desirable responding.

### 2.4 Data analysis

Preliminary analyses, reliability estimates, and correlations were computed in SPSS 26.0 (IBM Corp., Armonk, NY, United States). The hypothesized model was tested with the PROCESS macro (model 6; Hayes, 2013), which estimates serial mediation with ordinary least squares regression. Specific, total indirect, and contrast effects were estimated with 5,000 bias-corrected bootstrap resamples; confidence intervals that do not include zero were treated as significant. Demographic variables (gender, age, tenure) were treated as descriptive rather than as covariates, because the hypotheses concern theory-specified psychological mechanisms rather than demographic differences, none of the demographic variables showed a significant bivariate association with any study variable (all *p* > 0.05), and the decision was probed directly by re-estimating the model with the demographics included (see Results). Measurement quality was examined with confirmatory factor analysis of the four-factor structure in AMOS 26.0 (IBM Corp., Armonk, NY, United States), and model fit was judged against conventional cutoffs (Hu and Bentler, 1999). Discriminant validity was assessed with heterotrait-monotrait ratios, which remained at or below 0.551, far under the 0.85 benchmark (Henseler et al., 2015); construct validity criteria follow Fornell and Larcker (1981). All measures were self-reported, so common method variance was examined in two complementary ways beyond the procedural remedies of anonymity, distinct scale anchors, and neutral framing: Harman's single-factor test, in which the first unrotated factor accounted for 33.10% of the variance, below the conventional 40% threshold, and a one-factor confirmatory model compared against the four-factor structure, on the ground that a single latent factor unable to reproduce the covariance structure cannot serve as a plausible method-bias account (Podsakoff et al., 2003, 2024).

## 3 Results

Table 1 presents the descriptive statistics and correlations for the study variables; regression estimates appear in Table 2 and indirect effects in Table 3.

**Table 1:** Descriptive statistics and correlations among study variables (*N* = 476).

Variable	M	SD	α	1	2	3	4
1. Ethical leadership	3.69	0.37	0.93	1	0.49	0.44	0.37
2. Organizational fairness	3.56	0.34	0.90	0.49	1	0.49	0.43
3. Organizational identification	3.69	0.41	0.87	0.44	0.49	1	0.47
4. Responsible initiative	3.63	0.38	0.89	0.37	0.43	0.47	1

*N* = 476. Diagonal values are 1. α = Cronbach's alpha. All correlations are significant at *p* < 0.01 (two-tailed).

**Table 2:** Serial mediation regression results (PROCESS model 6; *N* = 476).

Predictor	*b*	SE	*t*	*p*
Equation 1: organizational fairness (M1)				
Ethical leadership (X)	0.455	0.037	12.16	< 0.001
*R*^2^ = 0.238, F(1, 474) = 147.92^***^				
Equation 2: organizational identification (M2)				
Ethical leadership (X)	0.300	0.050	5.98	< 0.001
Organizational fairness (M1)	0.438	0.054	8.16	< 0.001
*R*^2^ = 0.294, F(2, 473) = 98.40^***^				
Equation 3: responsible initiative (Y)				
Ethical leadership (X)	0.139	0.048	2.90	0.004
Organizational fairness (M1)	0.235	0.053	4.44	< 0.001
Organizational identification (M2)	0.286	0.043	6.73	< 0.001
*R*^2^ = 0.288, F(3, 472) = 63.56^***^				
Total effect (Y on X alone)				
Ethical leadership (X)	0.389	0.044	8.77	< 0.001
*R*^2^ = 0.140, F(1, 474) = 76.94^***^				

*N* = 476.

^***^p < 0.001. Unstandardized coefficients are reported.

**Table 3:** Indirect effects of ethical leadership on responsible initiative (*N* = 476).

Pathway	Estimate	95% CI
Ethical leadership → organizational fairness → responsible initiative	0.107	[0.059, 0.162]
Ethical leadership → organizational identification → responsible initiative	0.086	[0.053, 0.127]
Ethical leadership → organizational fairness → identification → responsible initiative	0.057	[0.037, 0.086]
Total indirect effect	0.250	[0.192, 0.316]
Total effect	0.389	[0.301, 0.477]
Contrast: identification vs. fairness pathway	−0.021	[-0.090, 0.051]
Contrast: identification vs. serial pathway	0.029	[-0.008, 0.069]

*N* = 476. Bias-corrected bootstrap estimates based on 5,000 resamples; confidence intervals excluding zero indicate significance. Contrasts test pairwise differences between specific indirect effects.

Table 1 presents the descriptive statistics and correlations. Mean scores sat between the midpoint and the agree pole for all four constructs, indicating that cadres viewed their institutions as broadly well led but not uniformly so. Ethical leadership correlated positively with organizational fairness (*r* = 0.49), organizational identification (*r* = 0.44), and responsible initiative (*r* = 0.37); fairness and identification correlated at *r* = 0.49, and both correlated positively with responsible initiative. Internal consistency was acceptable for all scales.

The four-factor measurement model fit the data well, χ^2^(588) = 733.10, CFI =.982, TLI =.981, RMSEA =.023, SRMR =.036, supporting the distinctness of the four constructs; heterotrait-monotrait ratios for all construct pairs remained below 0.551. Harman's single-factor test left 33.10% of variance in the first factor, and a one-factor confirmatory model fit the data far worse than the four-factor model, χ^2^(594) = 3800.62, CFI =.612, with the fit difference significant, Δχ^2^(6) = 3067.54, *p* < 0.001, so common method variance is unlikely to account for the structural results. Regression estimates appear in Table 2.

In the first-stage equation, ethical leadership was positively associated with organizational fairness (a_1_ = 0.455, *t* = 12.16, *p* < 0.001), explaining 23.80% of its variance. In the second-stage equation, ethical leadership (a_2_ = 0.300, *t* = 5.98, *p* < 0.001) and organizational fairness (d_21_ = 0.438, *t* = 8.16, *p* < 0.001) were both positively associated with organizational identification, together explaining 29.40% of its variance. In the outcome equation, responsible initiative was positively associated with organizational fairness (b_1_ = 0.235, *t* = 4.44, *p* < 0.001) and organizational identification (b_2_ = 0.286, *t* = 6.73, *p* < 0.001), and ethical leadership retained a positive direct association (c′ = 0.139, *t* = 2.90, *p* =.004), with the equation explaining 28.80% of the variance. The total effect of ethical leadership on responsible initiative was 0.389 (*t* = 8.77, *p* < 0.001), supporting H1.

Table 3 reports the indirect effects. The indirect effect of ethical leadership on responsible initiative through organizational fairness was 0.107 [95% CI (0.059, 0.162)], supporting H2. The indirect effect through organizational identification was 0.086 [95% CI (0.053, 0.127)], supporting H3. The serial indirect effect through fairness followed by identification was 0.057 [95% CI (0.037, 0.086)], supporting H4. The three pathways jointly accounted for 64.2% of the total effect (0.389). Pairwise contrasts among the specific indirect effects did not reach significance [largest contrast = 0.029, 95% CI (-0.008, 0.069)], so the present data do not support a strength claim for any one pathway over the others.

Two sensitivity checks probed the stability of these estimates. Re-estimating the serial model with gender, age, and tenure as covariates left the three specific indirect effects essentially unchanged [fairness pathway = 0.108, 95% CI (0.057, 0.163); identification pathway = 0.087, 95% CI (0.053, 0.131); serial pathway = 0.057, 95% CI (0.037, 0.085)]. Adding institution-type indicators (public, private, vocational) to the covariate set likewise left the estimates essentially unchanged (fairness = 0.108, identification = 0.089, serial = 0.056). The reported estimates are therefore not an artifact of demographic composition or institutional mix.

## 4 Discussion

This study asked how ethical leadership relates to responsible initiative among university middle-level cadres, and through what psychological route. Four findings emerged. Ethical leadership was positively associated with responsible initiative; organizational fairness mediated part of this association; organizational identification mediated part of it as well; and the two operated in sequence, with fairness associated with stronger identification. Together the results are consistent with the proposed pathway, in which the exchange and identity processes described by social exchange theory and social identity theory statistically account for the association between ethical leadership and responsible initiative, and they do so in a population, Chinese university middle-level cadres, that proactive behavior research has rarely sampled.

The mediation findings fit the exchange logic closely. Ethical leadership was strongly associated with perceived organizational fairness, consistent with the view that fair treatment is the visible signature of ethical conduct in leadership positions (Brown et al., 2005; Colquitt, 2001), and the fairness-based indirect effect confirmed that cadres who feel fairly treated return discretionary effort to the institution (Blau, 1964; Cropanzano and Mitchell, 2005). The pattern matches meta-analytic links between justice perceptions and extra-role outcomes (Colquitt et al., 2001; Cohen-Charash and Spector, 2001) and extends them from general employee samples to administrative cadres. Because the direct association remained significant, ethical leadership likely also works through channels not measured here, such as felt trust or psychological safety, which have carried ethical leadership to voice in prior work (Walumbwa and Schaubroeck, 2009).

The identification route behaved as social identity theory predicts. Ethical leadership related to identification both directly and through fairness, and identification carried a substantial share of the association with responsible initiative, in line with the motivational consequences of organizational identification documented meta-analytically (Riketta, 2005) and with the group engagement model's claim that justice judgments build identity-based engagement (Tyler and Blader, 2003). Cadres who see the institution as fair appear to fold it into their self-definition (Ashforth and Mael, 1989; Mael and Ashforth, 1992), and once institutional fate becomes personal fate, its problems become personal business. This sequence gives the serial path a coherent theoretical reading: fairness is the evidence, identification is the bond, and responsible initiative is the behavioral return.

Read against the aims of this research topic, the findings speak to its call for mediating and moderating mechanisms in the family of ethical leadership and workplace equity constructs. Recent contributions have traced ethical leadership to employee wellbeing through emotional energy and moral signals (Chen et al., 2026) and to ethical climate under team-level leadership (Zhang et al., 2026); the present results add a proactive-behavior outcome and a dual-mediator route, and they echo evidence that Chinese university staff respond strongly to commitment-relevant pressures (Cheng et al., 2026). Collectivism was not measured and no cross-cultural comparison was conducted, so the present data cannot attribute this dual-route pattern to the collectivist Chinese context; the parallel operation of exchange and identity mechanisms is instead read as a contextually situated pattern whose cultural boundary conditions remain to be tested (Farh et al., 2004).

A further result deserves theoretical attention: none of the three indirect pathways differed significantly from the others. Non-significant contrasts do not establish equivalence, so we make no claim that the pathways are equally strong; the defensible conclusion is that the present data cannot rank them. What the pattern does suggest is that the exchange and identity routes are complementary rather than competing accounts of how ethical leadership relates to responsible initiative. Each route statistically accounted for an independent share of the association, and the significant serial path is consistent with the conversion proposed in the Introduction: the fairness-based exchange route also operated through identification, linking an exchange resource to an identity bond. For dual-process models of ethical leadership, the implication is that exchange and identity mechanisms are better modeled as cooperating channels whose joint operation, rather than their relative dominance, characterizes the association with proactive conduct.

The findings carry practical implications for university governance. For senior leaders, they identify the concrete behaviors through which influence over cadre initiative travels: distributive care in workload and reward allocation, transparent and consistent procedures, and respectful treatment in everyday interaction. Leadership development programs that rehearse these behaviors give more return than abstract ethics exhortation, because fairness perceptions, not ethics rhetoric, are what cadres act on. For administrative offices, appraisal and promotion systems are the other lever: procedures perceived as consistent and bias-free are associated with the very fairness judgments that accompany both reciprocity and identification. Evidence from workforce-policy implementation also suggests that poorly designed or implemented organizational arrangements may generate perceived discrimination among employees, underscoring the importance of fairness-sensitive personnel policies (Vali et al., 2016). Institutions that pair ethical leadership development with procedurally fair cadre management may be better placed to see a more self-starting middle stratum, pending experimental or longitudinal verification, which matters because middle managers remain the hinge on which university strategy turns (Wooldridge et al., 2008; Bryman, 2007).

Several limitations temper these conclusions. The cross-sectional design cannot establish temporal ordering, and although the model was grounded in theory, alternative orderings, such as initiative feeding back into fairness experiences, cannot be excluded; longitudinal or quasi-experimental designs would provide stronger causal evidence. All measures were self-reported, so common method variance cannot be ruled out despite the diagnostic and procedural remedies applied. Sampling was confined to one province and relied on convenience access through administrative meetings, limiting generalization to other regions and institution types, so the findings should be extended to other regions, countries, and higher education systems with caution. Collectivism was not measured, and cross-cultural designs that assess collectivism directly would clarify whether the dual-route pattern varies across cultural settings. The responsible-initiative measure was adapted for the cadre setting rather than drawn from a validated instrument, and its self-report format may overstate concordance with actual behavior. Model estimates are unadjusted for demographics, which were treated as descriptive rather than confounding, a decision the covariate sensitivity analysis in Results supports; effect sizes, while statistically reliable, were moderate in absolute terms, and conventions for their interpretation follow Cohen (1988). Respondents were nested within 11 institutions, but the anonymous design recorded no institution identifier, so institution-level clustering could not be modeled; the institution-type sensitivity analysis suggests this omission did not materially alter the estimates, and multi-center designs that record institutional membership would add value. Future work could pair cadres with their superiors in round-robin designs, measure initiative through archival indicators such as proposal submissions, and test boundary conditions, for example whether procedural fairness matters more than distributive fairness at different administrative ranks.

## Data Availability

The raw data supporting the conclusions of this article will be made available by the authors, without undue reservation.
